# Long non-coding RNA CIR inhibits chondrogenic differentiation of mesenchymal stem cells by epigenetically suppressing ATOH8 via methyltransferase EZH2

**DOI:** 10.1186/s10020-021-00272-9

**Published:** 2021-02-05

**Authors:** Feng Liu, De-Ye Song, Jun Huang, Hong-Qi Yang, Di You, Jiang-Dong Ni

**Affiliations:** 1 Department of Orthopedics, The Second Xiangya Hospital of Central South University, No.139 Renminzhong Road, Changsha, 410011 Hunan People’s Republic of China; 2 Department of Orthopedics, The Third Hospital of Changsha, Changsha, 410015 Hunan People’s Republic of China

**Keywords:** LncRNA CIR, ATOH8, Chondrogenic differentiation, EZH2, hUC-MSC, Osteoarthritis

## Abstract

**Background:**

Osteoarthritis (OA) is the most common articular disorder, leading to joint malfunction and disability. Although the incidence of OA is increasing globally, the treatment of OA is very limited. LncRNA CIR has been implicated in OA through unclear mechanisms. Here, we investigated the role of lncRNA CIR in chondrogenic differentiation.

**Methods:**

Human umbilical-cord-derived mesenchymal stem cells (hUC-MSCs) were obtained from human umbilical cords. Flow cytometry was used to analyze the surface markers of hUC-MSCs. Various culture conditions and corresponding staining assays were employed to assess the differentiation abilities of hUC-MSC. qRT-PCR, western blot, and immunostaining were used to measure expression levels of related genes and proteins such as lncRNA CIR, ATOH8, EZH2, and H3K27me3. RNA immunoprecipitation assay, biotin pull-down, and chromatin immunoprecipitaion assay were performed to analyze the interactions of lncRNA CIR, EZH2, H3K27me3 and ATOH8 promoter.

**Results:**

hUC-MSCs exhibited MSCs features and could differentiate into chondrocytes under specific conditions. LncRNA CIR was downregulated while ATOH8 was upregulated during the chondrogenic differentiation of hUC-MSCs. Knockdown lncRNA CIR or overexpression of ATOH8 promoted chondrogenic differentiation. Further, lncRNA CIR bound to EZH2 and repressed ATOH8 expression via EZH2-mediated H3K27me3, which promotes the methylation of ATOH8. Inhibition of ATOH8 reversed the effects of knockdown lncRNA CIR on chondrogenic differentiation.

**Conclusion:**

LncRNA CIR suppresses chondrogenic differentiation of hUC-MSCs. Mechanistically, lncRNA CIR could inhibit ATOH8 expression that functions to promote chondrogenic differentiation through EZH2-mediated epigenetic modifications.

## Introduction

Osteoarthritis (OA) is a very common but complex chronic degenerative disease characterized by degradations of articular cartilage, thickening of the joint capsule, and the formation of osteophyte, leading to the progressive loss of joint mobility and function (Mehl et al. 2018). It is estimated to be the fourth leading cause of disability in Asia and the incidence of OA worldwide is on a steady rise (Vina and Kwoh 2018; Fransen 2011). Currently, there are no effective treatments that can restore the function of joint (Vargas Negrin et al. 2014). The pathogenesis of OA is complicated and multifactorial, which is mainly associated with age-related loss of homeostatic balance (Martel-Pelletier 2016). Articular chondrocyte, the only cell type in the articular cartilage, which has the function of maintaining tissue homeostasis (Carballo et al. 2017). During OA development, processes like inflammatory responses and the release of reactive oxygen species (ROS) induce remarkable changes to articular chondrocytes that result in their malfunction and eventually death (Caldwell and Wang 2015; Lepetsos and Papavassiliou 2016). Therefore, understanding the molecular mechanisms of chondrocyte homeostasis is very important for the development of new therapeutic avenues.

Emerging evidence has shown that mesenchymal stem cells (MSCs) could be used for cartilage repair in that they are multipotent stromal cells and can differentiate into varieties of cell types, including chondrocytes (Chang et al. 2016; Ullah et al. 2015; Fahy et al. 2018). Bone marrow MSCs are the most common source of MSCs and can promptly boost the cartilage regeneration (Richardson 2016; Gugjoo et al. 2016). Nevertheless, the process to collect bone marrow MSCs is invasive and may cause potential infections to the donors (Lukomska 2019). Human umbilical-cord-derived MSCs (hUC-MSCs) are another attractive source that could be potentially used for OA treatment and they are easy to obtain and store (Ding et al. 2015; Wang 2018). However, the mechanisms underlying the chondrocyte differentiation of hUC-MSCs remains largely unknown.

Long non-coding RNAs (lncRNAs) are a class of noncoding RNAs that have been reported to play a critical role in a number of processes including physiological processes and pathological processes (Yao et al. 2019; DiStefano 2018). Changes in the expression of lncRNAs have been linked to many diseases, including cancers and OA (DiStefano 2018; Cen et al. 2017). For example, lncRNA MSR was reported to increase during OA while lncRNA H19 was diminished (Liu 2016; Steck 2012). Moreover, manipulating the expression levels of those lncRNAs could affect OA progression (Cen et al. 2017; Chen et al. 2018). Recently, lncRNA CIR was observed elevated during OA and inhibition of lncRNA CIR mitigated the progression of OA (Lu et al. 2018). However, the underlying mechanisms of how lncRNA CIR regulates chondrogenic differentiation are unclear.

Atonal homolog 8 (ATOH8), also known as Math6, is a transcription factor of the basic helix-loop-helix (bHLH) protein family (Inoue 2001). It is broadly expressed in many tissues such as the kidney, brain, and skeletal muscles, which plays a crucial role in the regulation of cell proliferation and differentiation (Chen 2011). Previous studies have indicated that ATOH8 regulates chondrocyte proliferation and differentiation via Indian hedgehog (Ihh) signaling (Schroeder et al. 2019). Further, reduced expression of transcription factors including ATOH8 have been observed in OA and these reductions are associated with enhanced epigenetic modifications (Alvarez-Garcia 2016). Interestingly, EZH2, a histone methyltransferase, has been implicated to repress ATOH8 expression via elevating H3K27 trimethlylation (H3K27me3) in hepatocellular carcinoma (Zhang 2019). Our initial RNA–protein interaction prediction (RPIseq) analysis suggests that lncRNA CIR might bind to EZH2. We thus hypothesized that lncRNA CIR might regulate chondrogenic differentiation through EZH2/ATOH8 pathway.

In the present study, we carried out a series of experiments (the flow chat showed in Additional file 1: Fig. S1) to investigate the function of lncRNA CIR in chondrogenic differentiation, with particular focuses on the regulation of EZH2/ATOH8 pathway. We found that lncRNA CIR was reduced while ATOH8 was increased during chondrogenic differentiation of hUC-MSCs. Both knockdown lncRNA CIR and ATOH8 overexpression facilitated chondrogenic differentiation. Further, lncRNA CIR could suppress ATOH8 expression by promoting EHZ-mediated H3K27 trimethylation, and silencing ATOH8 reversed the effects of lncRNA CIR knockdown on chondrogenic differentiation. Our study reveals an essential role of lncRNA CIR in chondrogenic differentiation and sheds light on the roles of hUC-MSCs in OA. These findings could provide some avenues for further OA therapeutic treatments.

## Materials and methods

### Human umbilical-cord-derived mesenchymal stem cells (hUC-MSCs) culture

Umbilical cords for hUC-MSCs preparation were obtained from healthy pregnant patients (24–28 years old) without infectious diseases in the Second Xiangya Hospital, Central South University (Changsha, China). The study was reviewed and approved by the Ethics Committee of the Second Xiangya Hospital, Central South University and a written informed consent was signed by each patient. Umbilical cord samples were cut into 1–2 mm^3^ pieces and then digested with 0.075% collagenase II (Sigma, USA) and 0.125% trypsin (Gibco, USA) at 37 °C for 45 min followed by a pass through the 100-μm filter. Cell suspensions were washed with PBS and then cultured in Dulbecco’s modified Eagle medium (DMEM)/F12 (1:1) (Thermo Fisher Scientific, USA) supplemented with 10% fetal bovine serum (FBS, Neuromics, USA), 1% penicillin–streptomycin (Gibco), 2 mM L-glutamine (Gibco), and 10 ng/mL epidermal growth factor (EGF; Sigma). The cells were maintained at 37 °C in a humidified atmosphere containing 5% CO_2_ and the medium was changed twice a week.

### hUC-MSC characterization

The phenotypes of hUC-MSCs were determined by flow cytometry. Briefly, the cells were detached with 0.05% trypsin–EDTA and washed with PBS twice when they grew up to 80–90% confluence. Cell suspensions were blocked with 1% goat serum for 30 min at room temperature followed by incubation with different fluorescent-labeled antibodies against CD34, CD44, CD45, and CD90 (1:500; BD Biosciences, USA) at 4 °C for 30 min. Cells were further analyzed using a FACS Calibur flow cytometer (Becton Dickinson).

### hUC-MSC differentiation in vitro

For adipogenic differentiation, cells were seeded at a density of 1 × 10^5^ cells/well in 6-well plates and grow up to 80% confluence. The medium was switched to adipogenic medium (DMEM supplemented with 10% FBS) containing 10 μM insulin, 0.5 μM isobutylmethylxanthine, 1 μM dexamethasone, and 60 μM indomethacin (Sigma) for 21 days with medium changed every 3 days. Oil red oranse (Sigma) staining was performed to analyzed the differentiated adipocytes.

For osteogenic differentiation, cells were seeded at a density of 1 × 10^5^ cells/well in 6-well plates and grow up to 80% confluence. The medium was switched to osteogenic medium (DMEM supplemented with 10% FBS) containing 10 mM β-glycerol phosphate, 50 μM ascorbate, 0.1 μM dexamethasone (Sigma) for 3 weeks. Alizarin red (Sigma) staining was performed to analyze differentiated osteocytes.

For chondrogenic differentiation, cells were seeded at a density of 1 × 10^5^ cells/well in 6-well plates and grow up to 80% confluence. The medium was switched to chondrogenic medium (high glucose DMEM supplemented with 10% FBS) containing 100 nM dexamethasone, 10 ng/ml TGF-β3, 50 ng/ml ascorbate-2-phosphate, and 1 mM sodium pyruvate (Sigma) for 21 days. Alcian blue (Sigma) staining was performed to analyze differentiated chondrocytes.

### Plasmid and cell transfection

si-CIR/pcDNA3.1-CIR, si-EZH2/pcDNA3.1-EZH2, si-ATOH8/pcDNA3.1-ATOH8 were purchased from GenePharma, Shanghai, China. Transfection was performed using Lipofectamine 3000 (Invitrogen, Missouri, USA) according to manufacturer’s instruction. Briefly, cells (1 × 10^5^ cells/well) were cultured in 6-well plates and grown until 80% confluence. 1 μg of plasmid was used for transfection together with 1 μL Lipofectamine 3000. Cells were subjected to differentiation immediately following transfection and then harvested at indicated time.

### Subcellular fractionation

Subcellular fractionation was performed as previously described (Tan et al. 2015, 2020). Briefly, the cells were homogenerated in homogeneration buffer [320 mM sucrose, 5 mM sodium pyrophosphate, 1 mM EDTA, 10 mM Hepes (pH 7.4)] supplemented with protease inhibitor (Roche). Homogenate was then centrifuged at 800 × *g* for 10 min at 4 °C to yield nuclei (pellet). Supernatant was collected and centrifuged at 20, 000 × *g* for 20 min to yield membrane fraction (pellet) and cytoplasm fraction (supernatant).

### RNA extraction and qRT-PCR

Total RNA was isolated from treated cells using Trizol reagent (Invitrogen) according to the manufacturer's instructions. DNaseI was added into the lysis buffer to avoid DNA contamination. Cytoplasmic and Nuclear RNA purification was perfumed using the purification kit (Norgen, Canada) according to the manufacturer’s protocol. 1 μg total RNA from each sample was subjected to reverse transcription using the SuperScript® IV First-Strand Synthesis System (Invitrogen) before PCR amplification using 1 × Power SYBR® Green PCR Master Mix (Invitrogen). Relative expression levels of lncRNA or mRNA were normalized to 18S RNA and GAPDH mRNA, respectively, as internal controls. The following primers were used in this study:

lncRNA CIR forward primer: 5′-ACACTTGCAAGCCTGGGTAG-3′

lncRNA CIR reverse primer: 5′-CCATTTTCCTGTTGGTGCGG-3′

ATOH8 forward primer: 5′-CCTGAGGATCGCCTGTAACT-3′

ATOH8 reverse primer: 5′-TGTCAAAGGCTCCGAAAAGT-3′

18S RNA forward primer: 5′- CAGGATTGACAGATTGATAGC TCT-3′

18S RNA reverse primer: 5′-GAGTCTCGTTCGT TATCGGAATTA-3′

GAPDH forward primer: 5′-CCAGGTGGTCTCCTCTGA-3′

GAPDH reverse primer: 5′-GCTGTAGCCAAATCGTTGT-3’

### Western blotting

Total protein was extracted from treated cells with RIPA lysis buffer (Beyotime Institute of Biotechnology, Nantong, China) and the concentration of the protein was determined with Pierce™ BCA Protein Assay Kit (Thermo Fisher Scientific). Protein samples were separated by 12% SDS–polyacrylamide gels and then transferred to PVDF membranes (EMD Millipore, USA). The membranes were blocked for 1 h at room temperature. Subsequently, primary antibodies were added and incubated overnight at 4 °C. Following washes with TBST, the membranes were incubated with a secondary antibody for 1 h at room temperature. Protein bands were visualized using the ECL kit. The following primary antibodies were used: Anti-ATOH8 (1:1000; Abcam, USA); Anti- sex determining region Y-box 9 (SOX9) (1:1000; Abcam); Anti-Aggrecan (1:500; Abcam); Anti-type-II collagen (Col2A1) (1:500; Santa Cruz, USA); Anti-EZH2 (1:1000; Cell Signaling Technology, USA); Anti-H3K27me3 (1:1000; Abcam); Anti-GAPDH (1:10,000; Abcam); Anti-β-actin (1:1000; Santa Cruz).

### RNA Immunoprecipitation (RIP) assay

Transfected cells were lysed in lysis buffer (50 mM Tris–HCl, 150 mM NaCl, 2 mM EDTA, 1% NP-40, 0.5% sodium deoxycholate) containing RNase inhibitors and protease inhibitors (Thermo Scientific, Waltham, MA, USA). The extracted protein was incubated with relevant antibodies (anti-EZH2, 1:1 000 dilution and IgG as control) (Abcam) overnight at 4 °C and then pulled down with protein G Sepharose 4 Fast Flow suspension (GE Amersham, Little Chalfont, UK). The beads were digested with proteinase K (Sangon, Shanghai, China) for 1 h followed by RNA purification with Trizol reagent (Invitrogen). Quantitative RT-PCR was performed to examine the RNA yield.

### Biotin pull-down assay

The biotinylated lncRNA CIR was prepared using the MEGshortscript™ T7 kit based on the manufacturer’s protocol and then incubated with cell lysates from hUC-MSCs at 4 °C for 1 h followed by mixture with streptavidin-coupled dynabeads (Invitrogen, Shanghai, China) at 4 °C for another 3 h. The beads were then washed with TENT (10 mM Tris–HCl [pH 8.0], 1 mM EDTA [pH 8.0], 250 mM NaCl, 0.5% Triton X-100) buffer 3 times and eluted with 1 × laemmli SDS sample buffer. The elution was heated for 5 min at 95 °C and proceeded to western blotting analysis of EZH2 and H3K27me3.

### Chromatin immunoprecipitation (ChIP) assay

ChIP was performed using the commercial ChIP kit (Cell Signaling Technology) according to the manufacturer’s protocol. For each chromatin immunoprecipitation, 5 μg of anti-EZH2 or anti-H3K27me3 antibodies and 1 μL of normal IgG were used. After immunoprecipitation, chromosomal DNA was purified. ATOH8 promoter region was detected by using PCR. The primers used for amplifying the ATOH8 promoter region were as follows: forward: 5′-GCGTGACTTTGGAGCTTTCG-3′; reverse: 5′-ACTCGCCACGAGACAGAAAA-3′. The results were expressed as relative input.

### Immunostaining

Cells were fixed in 4% paraformaldehyde (PFA) at room temperature for 15 min and permeabilized with 0.1% Triton X-100 in PBS for half an hour at room temperature. Then cells were blocked with 1% BSA in PBS for 1 h at room temperature followed by incubation with primary antibodies at 4 °C overnight. Cells were then washed with PBS and incubated with secondary antibody conjugated with TRITC conjugated secondary antibody for 2 h at room temperature. Images were acquired with standard microscope.

### Statistical analysis

All cell experiments were performed at least three times and analyzed using Graphpad Prism 8. Statistical significance was determined by unpaired two-tailed Student’s *t* test for two groups or one-way ANOVA followed by *Tukey’s *post hoc* test* for multiple groups. *P < 0.05, **P < 0.01, ***P < 0.001. Data were presented as Mean ± SD (standard deviation).

## Results

### Isolation and identification of hUC-MSCs

**Fig. 1:**
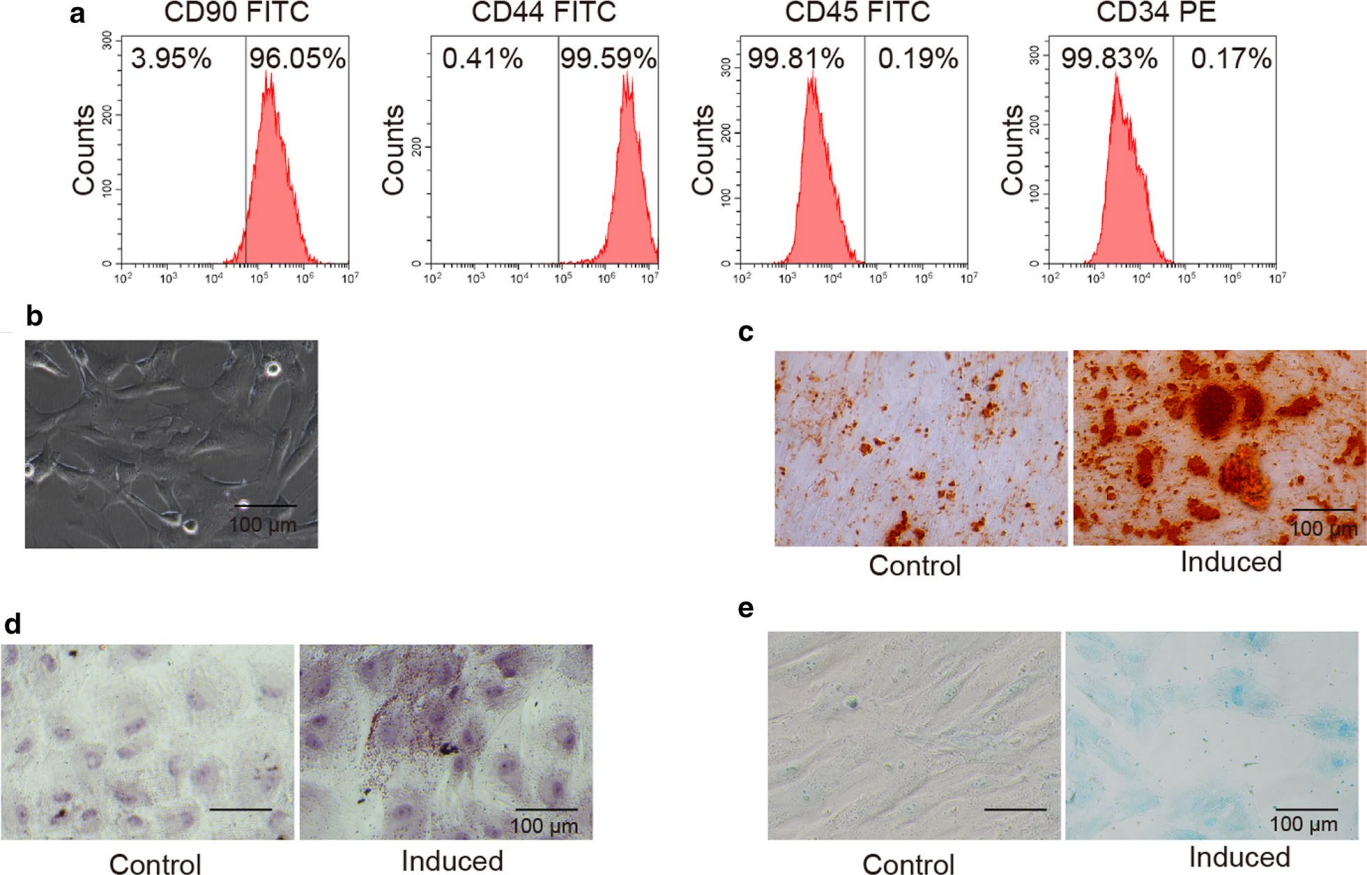
Isolation and identification of hUC-MSCs. **a** Flow cytometry analysis of surface expressions of CD34, CD44, CD45, and CD90. **b** Morphology of hUC-MSCs after 3 weeks’ culture. **c** Alizarin red staining of hUC-MSCs after 3 weeks’ culture in osteogenic medium. **d** Oil red oranse staining of hUC-MSCs after 3 weeks’ culture in adipogenic medium. **e** Alcian blue staining of hUC-MSCs after 3 weeks’ culture in chondrogenic medium

We first characterized the identities of hUC-MSCs. Human umbilical cord tissues were cultured for more than 2 weeks and spindle-shaped fibroblastic cells were observed Fig. 1b. MSCs are known to express CD13, CD29, CD44, CD90, CD105, but not CD34, CD45, CD117 (Ullah et al. 2015). Flow cytometry analysis revealed that our isolated hUC-MSCs were positive for CD44 and CD90 Fig. 1a, but negative for CD34 and CD45 signals, indicating that the cultured cells exhibited MSC features.

To characterize the differentiation potentials of hUC-MSCs, we cultured hUC-MSCs in various media including adipogenic, osteogenic, and chondrogenic medium for 3 weeks and then analyzed their differentiation abilities using correlated staining methods. Cells growing in osteogenic medium showed positive Alizarin red staining Fig. 1c, cells cultured in adipogenic medium exhibited positive oil red oranse staining Fig. 1d, and cells in chondrogenic medium revealed Alcian blue signal Fig. 1e. Together, these results demonstrate that our hUC-MSCs could differentiate into chondrocytes under specific conditions.

### LncRNA CIR knockdown promotes chondrogenic differentiation of hUC-MSCs

**Fig. 2:**
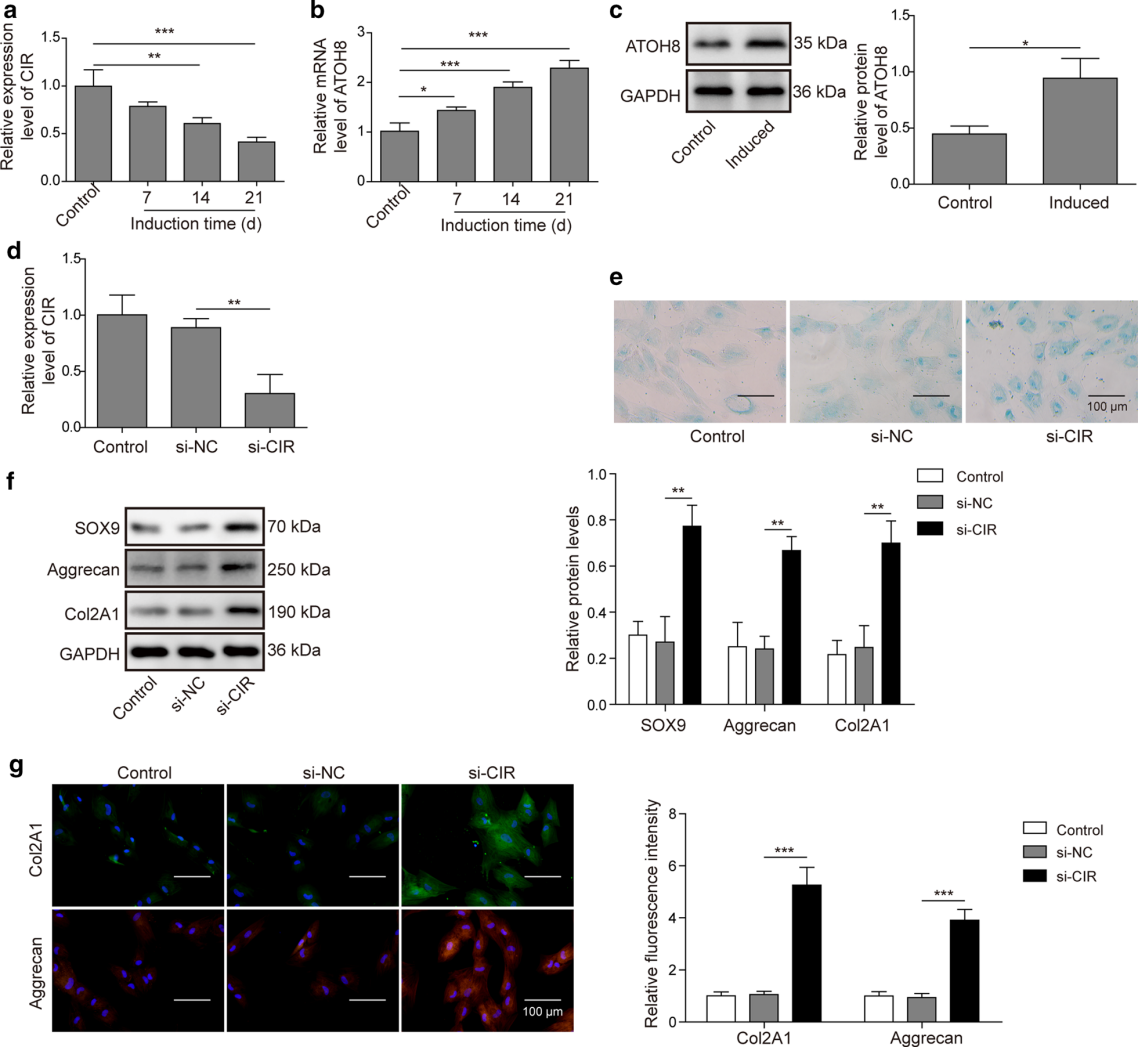
LncRNA CIR knockdown promotes chondrogenic differentiation of hUC-MSCs. **a** qRT-PCR analysis of lncRNA CIR level in control and differentiated hUC-MSCs. **b** qRT-PCR analysis of ATOH8 mRNA level in control and differentiated hUC-MSCs. **c** Western blot analysis of ATOH8 protein level in control and differentiated hUC-MSCs. **d** qRT-PCR analysis of lncRNA CIR level in different transfected hUC-MSCs. **e** Alcian blue staining to analyze chondrogenic differentiation of hUC-MSCs transfected with different constructs. **f** Western blot analysis of levels of chondrogenic markers including SOX9, Aggrecan, and Col2A1 in transfected cells. **g** Immunostaining analysis of levels of Col2A1 and Aggrecan in transfected cells. n = 3; *P < 0.05; **P < 0.01; ***P < 0.001

To examine the role of lncRNA CIR in chondrogenic differentiation, we first measured the level of lncRNA CIR during chondrogenic differentiation. With hUC-MSCs, we observed that lncRNA CIR was significantly reduced Fig. 2a, while both mRNA level and protein level of ATOH8 were elevated during chondrogenic differentiation Fig. 2b, c. Next, we manipulated lncRNA CIR level through siRNA and examined how it affected the chondrogenic differentiation. Cells transfected with si-CIR had much lower level of lncRNA CIR compared with control cells (non-transfected cells) or cells transfected with si-NC Fig. 2d. Interestingly, there were more Alcian blue positive cells when lncRNA CIR was knocked down through si-CIR compared with control cells, although the change was not very strong Fig. 2e. Moreover, hUC-MSCs transfected with si-CIR had higher levels of chondrogenic markers, such as SOX9, Aggrecan, Col2A1 than non-transfected cells or cells transfected with si-NC Fig. 2f. Immunostaining method also revealed higher signals of Col2A1 and Aggrecan in si-CIR treated cells Fig. 2g. Therefore, we conclude that lncRNA CIR knockdown could promote chondrogenic differentiation of hUC-MSCs evidenced by increase of chondrogenic marker expression.

### Overexpression of ATOH8 promotes chondrogenic differentiation of hUC-MSCs

**Fig. 3:**
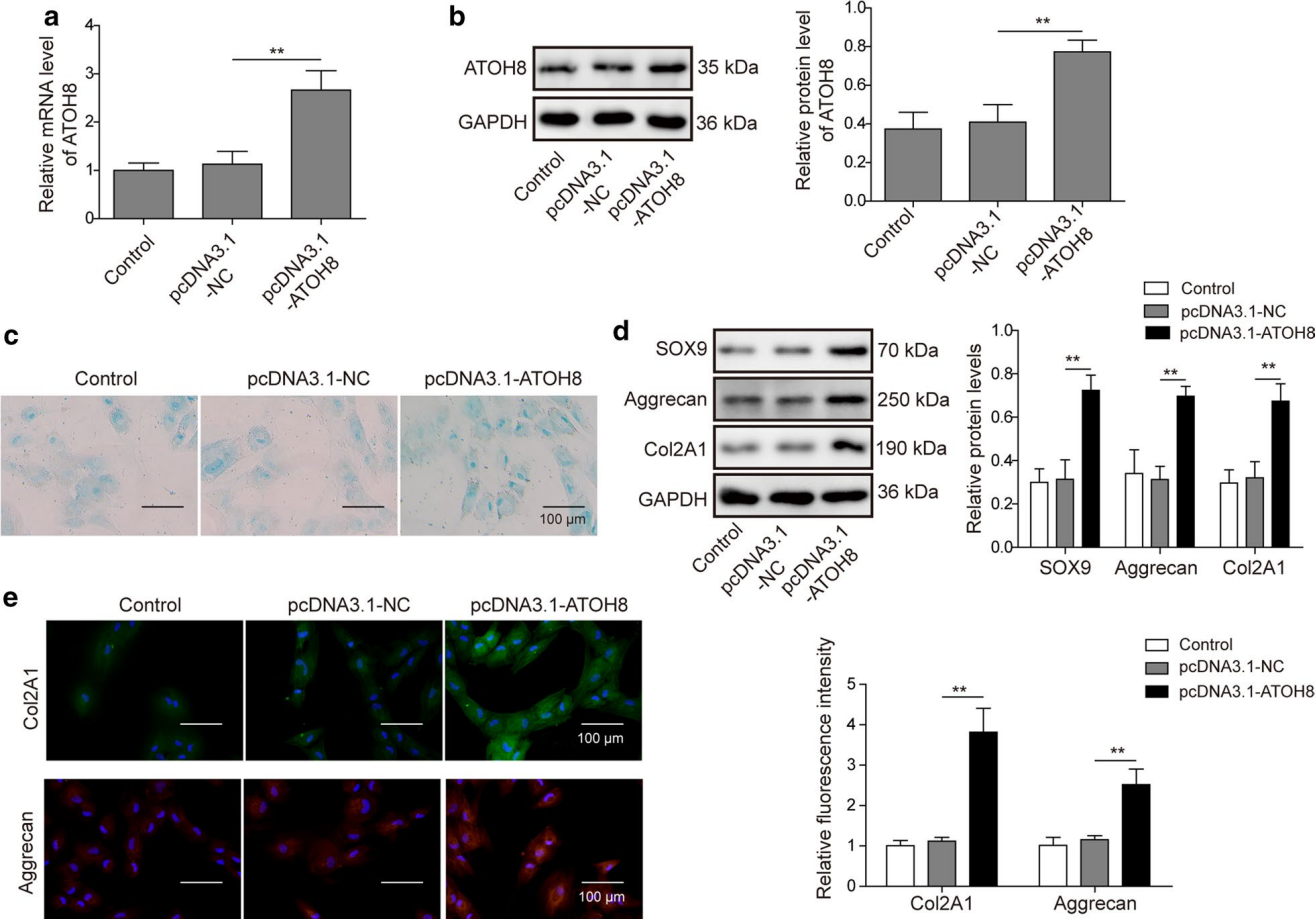
Overexpression of ATOH8 promotes chondrogenic differentiation of hUC-MSCs. **a** qRT-PCR analysis of ATOH8 level in transfected cells. **b** Western blot analysis of ATOH8 protein level in transfected cells. **c** Alcian blue staining to analyze chondrogenic differentiation of hUC-MSCs transfected with different constructs. **d** Western blot analysis of expression levels of SOX9, Aggrecan, and Col2A1 in transfected cells. **e** Immunostaining analysis of levels of Col2A1 and Aggrecan in transfected cells. n = 3; * *P* < 0.05; **P < 0.01; ***P < 0.001

We then investigated how ATOH8 regulated chondrogenic differentiation. Ectopic expression of pcDNA3.1-ATOH8 resulted in higher levels of ATOH8 mRNA, as well as ATOH8 protein Fig. 3a, b. We found that, similar to lncRNA CIR knockdown, increasing ATOH8 level had a small tendency to up-regulate the number of Alcian blue positive cells Fig. 3c. In addition, expressions of chondrogenic markers including SOX9, Aggrecan, and Col2A1 were all elevated by ATOH8 overexpression Fig. 3d, e. Taken together, these data show that ATOH8 may be a promoter in chondrogenic differentiation of hUC-MSCs.

### LncRNA CIR directly binds to EZH2

**Fig. 4:**
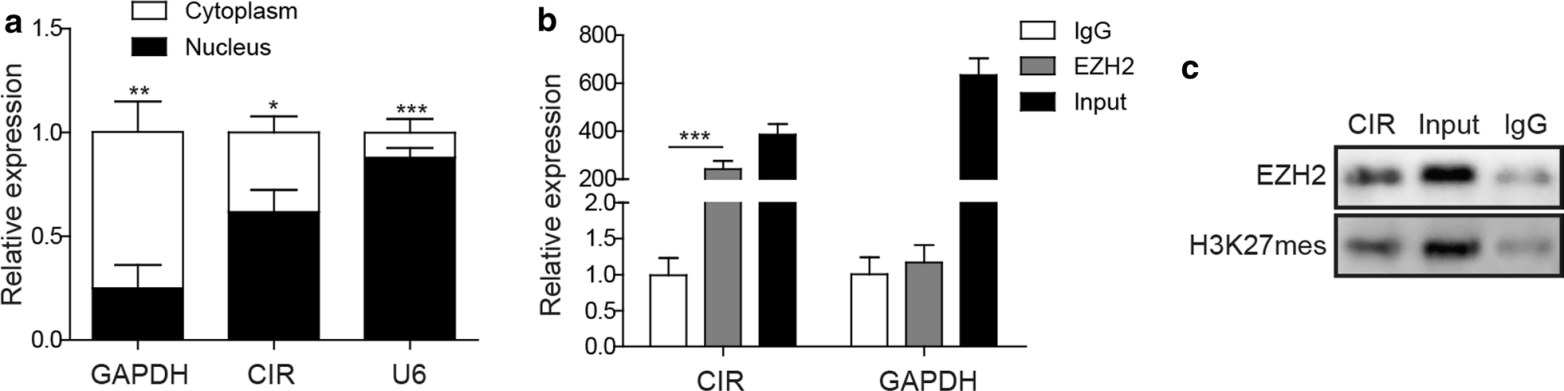
LncRNA CIR directly binds to EZH2. **a** qRT-PCR analysis of lncRNA CIR level in cytoplasm and nucleus. **b** RIP assay was performed to analyze lncRNA CIR/EZH2 interaction. **c** Biotin-RNA pull down assay was used to measure the interactions of lncRNA CIR with EZH2 and H3K27me3. n = 3; *P < 0.05; **P < 0.01; ***P < 0.001

We next sought to examine the regulatory relationship between lncRNA CIR and ATOH8, since they both regulated chondrogenic differentiation. EZH2, a histone methyltransferase, is the functional enzymatic component of the Polycomb Repressive Complex 2 (PRC2) (Tan et al. 2014). Changes of EZH2 directly affect the thrimethylation of H3K27 (H3K27me3) and methlylation of CpG islands (Pan et al. 2018), leading to alternations of many proteins, including ATOH8 (Zhang 2019). With some preliminary RPIseq analysis, we found that lncRNA CIR might bind to EZH2 (data not shown). To confirm this prediction, we first looked at subcellular localization of lncRNA CIR as EZH2 is predominantly localized to nucleus. With subcellular fractionation, as expected, we found that GAPDH was mainly localized in cytoplasm while U6 was predominantly expressed in nucleus Fig. 4a, indicating that we successfully isolated cytoplasm fraction and nucleus fraction. Regarding lncRNA CIR, we showed that the majority of lncRNA CIR was in the nucleus Fig. 4a, which is similar to EZH2. Using RIP assay, we found that immunoprecipitation with EZH2 antibody successfully pulled down lncRNA CIR Fig. 4b, indicating that lncRNA CIR binds to EZH2. Recent studies suggest that EZH2 represses ATOH8 expression via regulating methylation of its promoter and H3K27 trimethylation (Zhang 2019). We next performed RNA precipitation with a biotinylated lncRNA CIR probe and the results showed specific enrichments of EZH2 and H3K27me3 in the precipitates of lncRNA CIR Fig. 4c, indicating that lncRNA CIR binds to EZH2 and H3K27me3.

### LncRNA CIR binds to EZH2 and silences ATOH8 expression

**Fig. 5:**
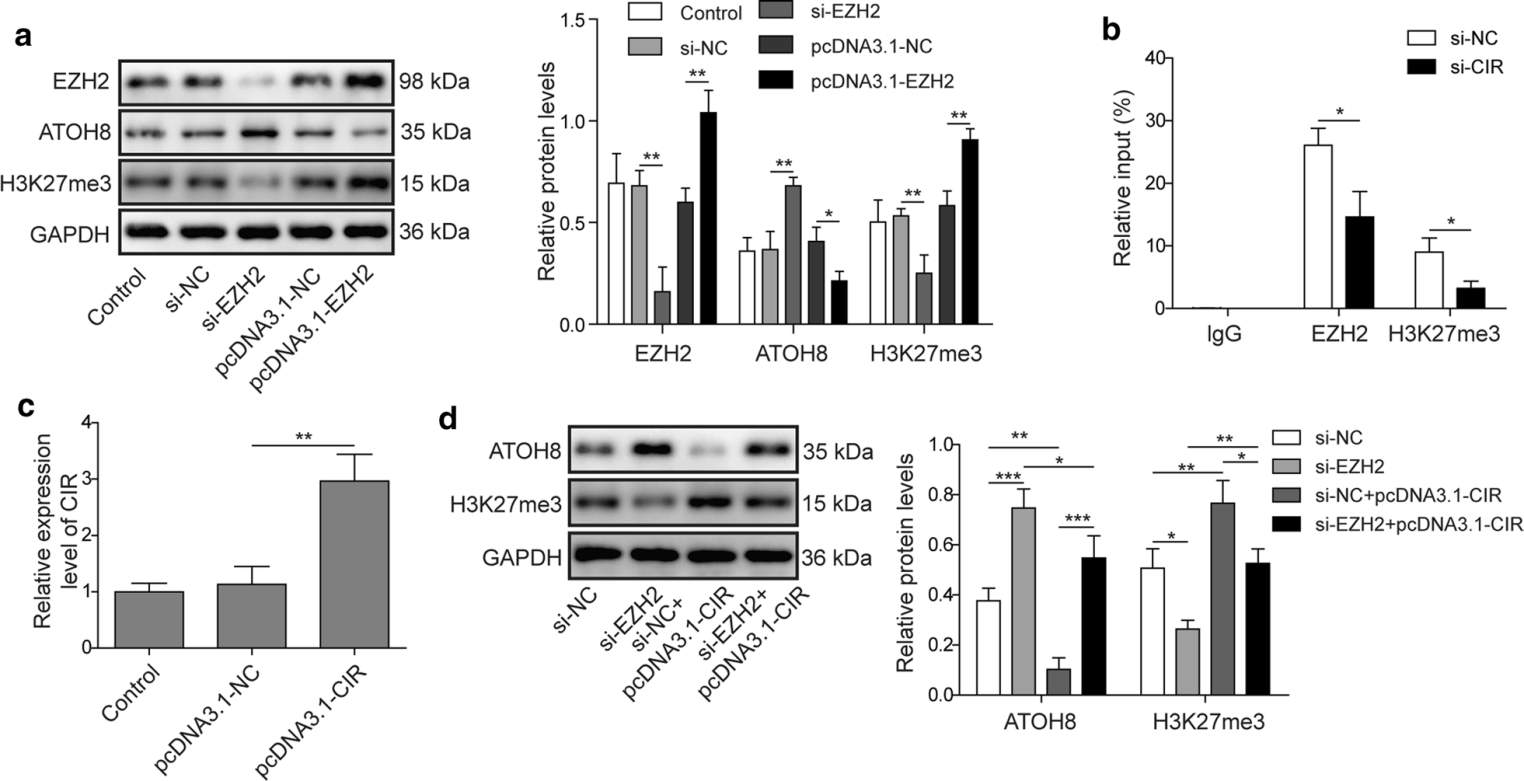
LncRNA CIR binds to EZH2 and silences ATOH8 expression. **a** Western blot analysis of EZH2, ATOH8 and H3K27me3 in transfected cells. **b** ChIP analysis was performed to analyze the interactions of EZH2 and H3K27me3 with ATOH8 promoter in transfected cells. **c** qRT-PCR analysis of lncRNA CIR level in transfected cells. **d** Western blot analysis of protein levels of ATOH8, H3K27me3 in transfected cells. n = 3; *P < 0.05; **P < 0.01; ***P < 0.001

The results presented above suggest that lncRNA CIR might regulate ATOH8 expression via binding to EZH2. We next directly examined that regulation. Knockdown EZH2 through si-EZH2 significantly diminished the levels of EZH2 and H3K27me3 but upregulated the level of ATOH8, while EZH2 overexpression showed the opposite trends Fig. 5a. The ATOH8 promoter is located in a typical CpG island and EZH2 suppresses ATOH8 expression through raising methylation of its promoter (Zhang 2019). Through ChIP-qRT-PCR, we found that silencing lncRNA CIR significantly weakened the bindings of EZH2 and H3K27me3 to ATOH8 promoter Fig. 5b, suggesting that lncRNA CIR promotes ATOH8 methylation. Further, EZH2 knockdown upregulated ATOH8 expression and downregulated H3K27me3 level, but overexpression of lncRNA CIR reversed the effects of EZH2 knockdown Fig. 5c, d. All together, these results demonstrate that lncRNA CIR could repress ATOH8 expression via promoting EZH2-mediated methylation.

### LncRNA CIR inhibits chondrogenic differentiation by epigenetically inhibiting ATOH8

**Fig. 6:**
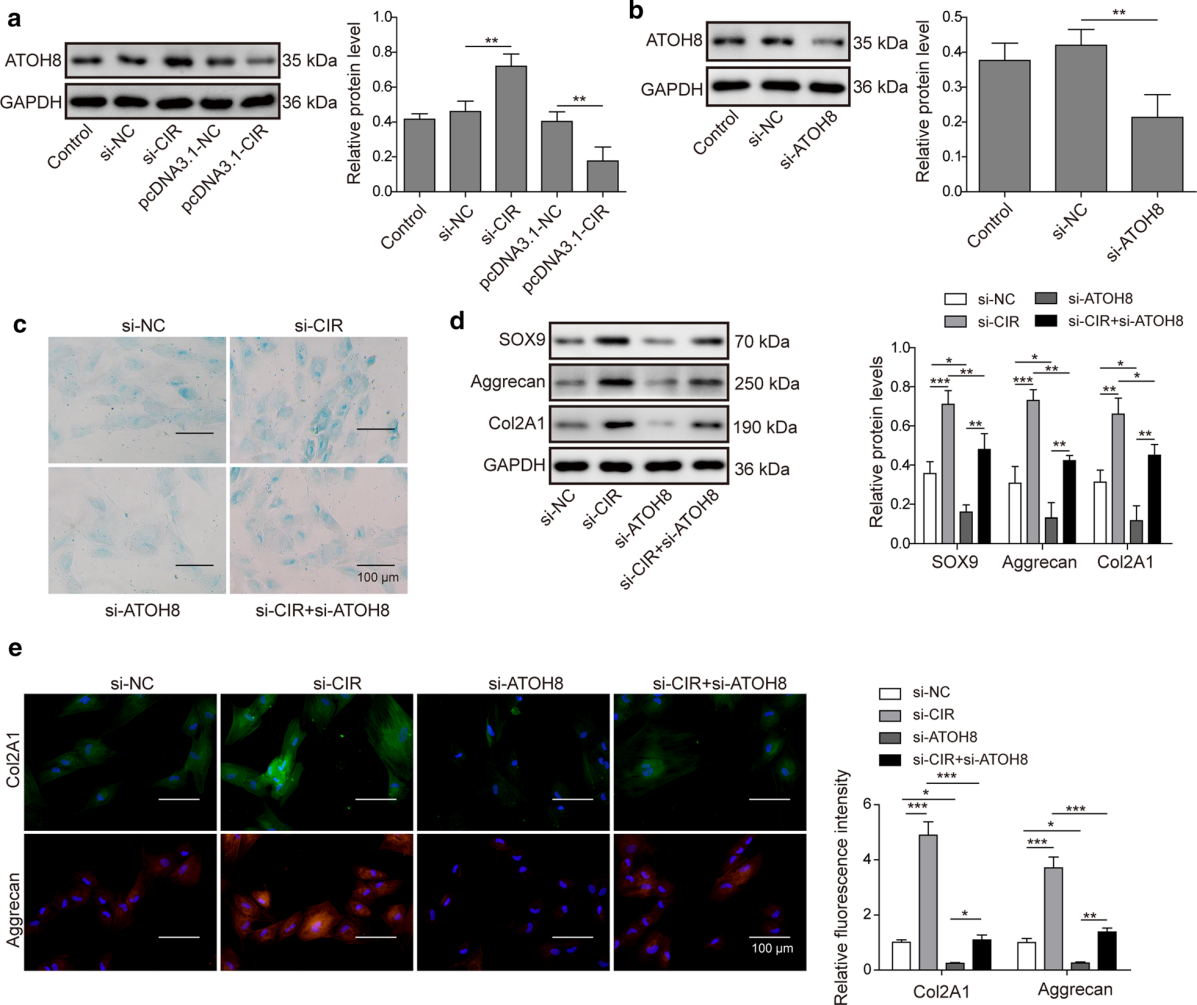
LncRNA CIR inhibits chondrogenic differentiation by epigenetically inhibiting ATOH8. **a** qRT-PCR analysis of ATOH8 mRNA levels in transfected cells. **b** Western blot analysis of ATOH8 protein levels in transfected cells. **c** Alcian blue staining to analyze chondrogenic differentiation of hUC-MSCs transfected with different constructs. **d** Western blot analysis of expression levels of SOX9, Aggrecan, and Col2A1 in transfected cells. **e** Immunostaining analysis of levels of Col2A1 and Aggrecan in transfected cells. n = 3; *P < 0.05; **P < 0.01; ***P < 0.001

In the end, we sought to reveal the mechanisms of how lncRNA CIR regulates chondrogenic differentiation of hUC-MSCs. Consist with aforementioned results, knockdown lncRNA CIR in hUC-MSCs increased ATOH8 level while overexpression of lncRNA CIR decreased Fig. 6a. Silencing ATOH8 mRNA through si-ATOH8 greatly reduced ATOH8 protein level as well Fig. 6b. Consistently, knockdown lncRNA CIR facilitated Alcian blue positive cells and silencing ATOH8 suppressed that, but these differences were not clear Fig. 6c. However, cells transfected with si-CIR had higher expressions of chondrogenic markers including SOX9, Aggrecan and Col2A1 while cells transfected with si-ATOH8 had lower levels compared to control cells Fig. 6d, e. Moreover, si-ATOH8 inhibited those upregulations mediated by lncRNA CIR knockdown Fig. 6d, e, suggesting that lncRNA CIR may suppress chrondrogenic differentiation through down-regulation of ATOH8.

## Discussion

OA is a chronic disorder caused by degeneration of the joint cartilage, leading to joint malfunction, pain, and disability (Vina and Kwoh 2018; Martel-Pelletier 2016). The prevalence is very high and the incidence of OA is progressively increasing, as it is highly associated with age (Vina and Kwoh 2018). However, the underlying mechanisms of OA development and progression are elusive. In the present study, we investigated the function of lncRNA CIR in OA with particular focus on the chondrogenic differentiation of hUC-MSCs. We observed that lncRNA CIR was decreased but ATOH8 was increased during chondrogenic differentiation. Knockdown lncRNA CIR may increase ATOH8 expression via repressing EZH2-mediated methylation and promoted chondrogenic differentiation. These results demonstrated an essential role of lncRNA CIR/EZH2/ATOH8 in chondrogenic differentiation, which could serve as a potential target for OA therapeutic development.

Chondrocytes are the only cells in the cartilage and are responsible for maintaining tissue homeostasis in the cartilage (Akkiraju and Nohe 2015). OA is characterized by degenerative chondrocytes (Singh et al. 2019). Previous studies have identified a huge number of hypermethylated transcription factors including ATOH8 in OA wherein the functions of chondrocytes are compromised, resulting in reduced expressions (Alvarez-Garcia 2016). In addition, lncRNA CIR has been shown elevated in OA cartilage (Lu et al. 2018). Here, we observed opposite changes of ATOH8 and lncRNA CIR during chondrogenic differentiation of hUC-MSCs. These results are very consistent and together with other studies (Schroeder et al. 2019; Alvarez-Garcia 2016; Wang et al. 2018; Liu 2014), supporting the model that lncRNA CIR inhibits chondrogenic differentiation while ATOH8 facilitates chondrogenic differentiation. Therefore, restoring the expression of ATOH8 or inhibition of lncRNA CIR could promote the differentiation of chondrocytes evidenced by expression of chondrogenic markers including SOX9, Aggrecan and Col2A1 and retard the development or progression of OA. Notably, accumulating evidence indicates that non coding RNAs play crucial roles in the OA, including lncRNAs and miRNAs, and that they could serve as therapeutic targets (Sun 2019). For instance, suppressing lncRNA HOTAIR could inhibit the cartilage degradation during OA (Yang 2020). It will be interesting to study the functions of other non-coding RNAs in OA.

LncRNAs are long non-coding RNAs that play important roles in regulating gene expressions during various cellular processes (Yao et al. 2019). Many lncRNAs have been reported to function as sponges of miRNA, which results in dis-inhibition of target gene expression (Zhang et al. 2018, 2016; Yue 2019). Here, we found lncRNA CIR regulated ATOH8 expression via a distinct mechanism. lncRNA CIR could bind to EZH2 and promotes EZH2-mediated H3K27 trimethylation of ATOH8, leading to reduced expression of ATOH8 protein. This finding suggests that lncRNA can modulate gene expressions through diverse mechanisms. Indeed, many other studies have demonstrated that lncRNAs directly interact with proteins involved in the regulation of chromatin conformation, indicating the interplay between lncRNA and epigenetic machinery (Neve et al. 2018; Morlando and Fatica 2018; Wang 2017; Han and Chang 2015). There may be other mechanisms underlying the effects of lncRNA CIR on ATOH8 expression and it might be interesting for future study.

hUC-MSCs are the very attractive avenue for regenerative medicine compared to bone marrow MSCs (Ding et al. 2015; Detamore 2013). Consistent with previous studies, we demonstrated that hUC-MSCs have great differentiation potential and can be differentiated into various types of cells with specific conditions, including chondrocytes (Wang 2018; Zhao 2019). Moreover, our work reveals a key role of lncRNA CIR/EZH2/ATOH8 signaling in chondrogenic differentiation process. Since our study is limited by in vitro cell culture system, it is very necessary to confirm these results with in vivo animal models. Additionally, three-dimensional (3D) cell culture system is a very useful in vitro cell model for diseases (Langhans 2018). It will be interesting to further study the role of lncRNA CIR/EZH2/ATOH8 signaling in chondrogenic differentiation with 3D cell model.

**Fig. 7:**
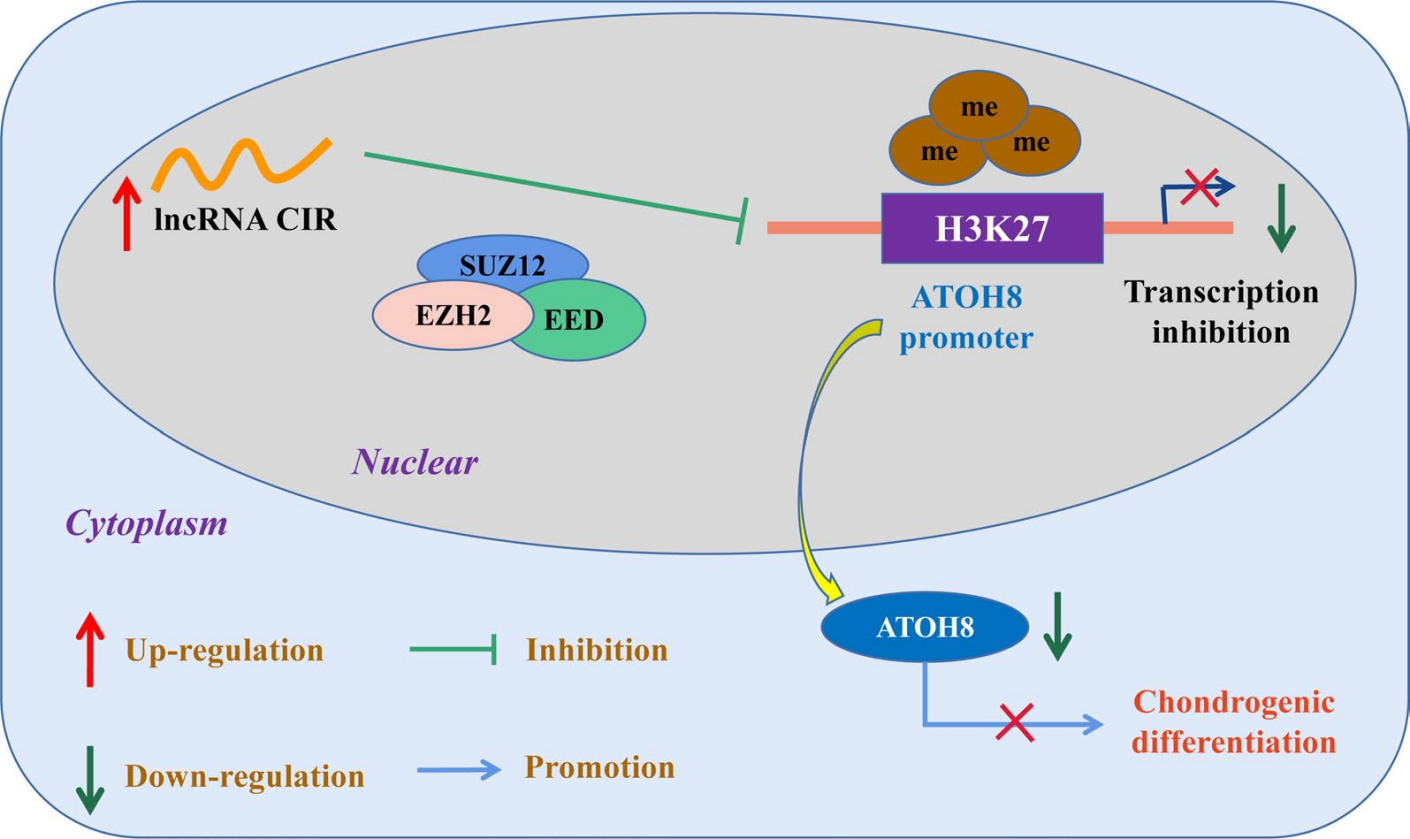
Schematic diagram demonstrates the role of lncRNA CIR/EZH2/ATOH8 axis in chondrogenic differentiation

In summary, our work presented here demonstrates that lncRNA CIR may regulate chondrogenic differentiation of hUC-MSCs via EZH2/ATOH8 pathway Fig. 7. Targeting lncRNA CIR/EZH2/ATOH8 pathway could be a potential avenue for future OA therapeutic development.

## Supplementary Information

**Additional file 1:** **Figure S1.** The flow chart of experimental procedure in this study.

## Data Availability

All data generated or analyzed during this study are included in this published article.
