# Supplementary figures and images for: Long non-coding RNA CIR inhibits chondrogenic differentiation of mesenchymal stem cells by epigenetically suppressing ATOH8 via methyltransferase EZH2
Source: Mol Med. 2021 Feb 5;27:12. doi: 10.1186/s10020-021-00272-9 (PMC7866678; doi:10.1186/s10020-021-00272-9)

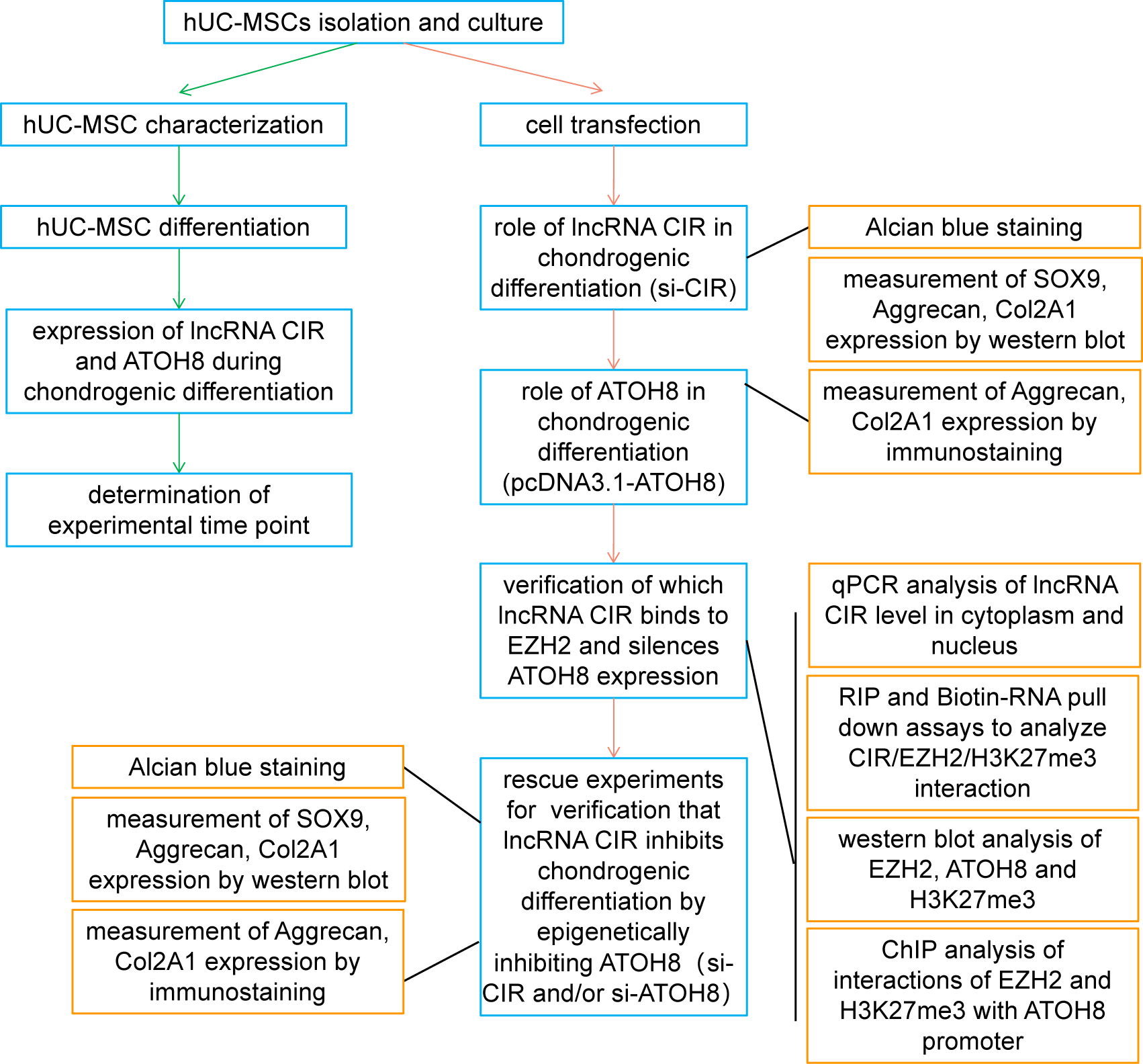

Supplement: Supplementary file 1 — Additional file 1: Figure S1. The flow chart of experimental procedure in this study. [file 10020_2021_272_MOESM1_ESM.tif]
